# Knowledge, attitudes, and practices of pharmacists toward over-the-counter medication use in Iraq: a nationwide cross-sectional study

**DOI:** 10.3389/fphar.2025.1669176

**Published:** 2025-12-02

**Authors:** Mohamed AbdElrahman, Mohammed D. Al-Rekabi, Ghadah Ali Al-Oudah, Aysam M. Fayed, Hasanain Kamil Owadh

**Affiliations:** 1 Clinical Pharmacy Department, College of Pharmacy, Al-Mustaqbal University, Hilla, Iraq; 2 Clinical Pharmacy Department, Badr University Hospital, Faculty of Medicine, Helwan University, Cairo, Egypt; 3 College of pharmacy, University of Alkafeel, Najaf, Iraq; 4 Molecular Biology Department, Genetic Engineering and Biotechnology Research Institute, University of Sadat City, Sadat City, Egypt; 5 Medical Laboratory Techniques Department, College of Health and Medical Technique, Al-Mustaqbal University, Babylon, Iraq

**Keywords:** over-the-counter medications, self medication, pharmacy practice, pharmacovigilanc, patient safety

## Abstract

**Introduction:**

Over-the-counter (OTC) medications play a vital role in global healthcare systems by offering accessible treatment options for minor ailments. However, the growing use of OTC drugs in Iraq raises concerns regarding pharmacists’ knowledge, safety perceptions, and dispensing practices.

**Methods:**

A nationwide cross-sectional survey was conducted among 529 licensed pharmacists from January to March 2025 across all major Iraqi regions. The questionnaire assessed demographics, familiarity with OTC drugs, safety attitudes, and dispensing behaviors.

**Results:**

Most participants (95.4%) reported familiarity with OTC medications and frequent encounters with self-medicating patients, particularly in the Central and Southern regions. NSAIDs were the most commonly recommended OTC category (71.8%). Significant regional differences were observed in familiarity, frequency of self-medication, and safety perceptions (p = 0.0071, 0.00088, and 0.045, respectively). Pharmacists with less than 5 years of experience were more likely to report adverse drug reactions (p = 0.0332) and to inquire about patients’ OTC use (p = 0.1035). Overall, younger pharmacists and those practicing in the Kurdistan region demonstrated greater familiarity with OTC medicines, while neutral safety perceptions and practice in Southern Iraq were associated with lower familiarity.

**Conclusion:**

Iraqi pharmacists demonstrate generally strong awareness and proactive attitudes toward OTC medication use. Nonetheless, gaps remain in adverse reaction reporting and drug interaction recognition. Implementing standardized continuing education and structured OTC safety training could enhance practice consistency and promote safer self-medication behaviors nationwide.

## 1 Introduction

Over-the-counter (OTC) drugs constitute a substantial segment of the worldwide pharmaceutical business. (Sánchez-Sánchez et al., 2021). The availability of OTC medications, along with their convenience and cost-effectiveness, renders them a vital element of global healthcare systems (Bigdeli et al., 2013; Yenet et al., 2023). The rising utilization of over-the-counter pharmaceuticals in Iraq, similar to numerous other nations, has elicited apprehensions about their proper usage and the responsibility of pharmacists in guaranteeing the safe and effective distribution of these drugs (Data and Use, 2020). Pharmacists serve as the initial point of contact in numerous healthcare environments, playing a vital role in educating patients and offering information on the appropriate utilization of over-the-counter medications (Andy et al., 2024; Ilardo and Speciale, 2020).

Pharmacists, as healthcare professionals, are required to possess an extensive knowledge of drug interactions, adverse effects, and appropriate utilization of over-the-counter pharmaceuticals (Andy et al., 2024; Ansari, 2010; Hughes et al., 2002). This understanding is essential for preventing usage, minimizing adverse drug reactions, and improving patient safety (Cullen et al., 2000; Benjamin, 2003). Furthermore, pharmacists’ views and behaviors regarding OTC drugs might profoundly impact public health results. The growing dependence on self-medication has rendered the job of pharmacists in distributing OTC medications and providing recommendations more pivotal than ever (Akande-Sholabi and Akinyemi, 2023). Nonetheless, despite their significance in medication management, the knowledge, attitudes, and behaviors of pharmacists concerning OTC drug usage in Iraq have not been thoroughly examined (Hatem et al., 2024).

Studies indicate that pharmacists’ understanding of OTC medications significantly differs among various locations and healthcare systems. In numerous nations, pharmacists frequently lack enough preparation for the issues presented by the increasing demand for over-the-counter pharmaceuticals (Ravichandran et al., 2016; Feng et al., 2022). Research across many environments has revealed deficiencies in pharmacists’ comprehension of OTC drug interactions, associated hazards, and the effective communication of these concerns to patients (Seubert et al., 2018). Moreover, although numerous nations have formulated guidelines for the distribution of OTC medications, the actual practices of pharmacists may not consistently conform to these recommendations, potentially resulting in public health hazards (Jairoun et al., 2023).

A study in Saudi Arabia indicated that while pharmacists had moderate knowledge of OTC drugs, their patient counseling techniques were insufficient, frequently resulting in inappropriate self-medication behaviors (Jalal and Jalal, 2024). Comparable outcomes have been noted in other Middle Eastern nations, suggesting that the problem of insufficient knowledge and substandard dispensing procedures is not exclusive to Iraq (Obaid et al., 2022). Moreover, research in Western nations has demonstrated that even within robust healthcare systems, the comprehension and administration of OTC medications can be variable, warranting deeper exploration of the factors that affect pharmacists’ practices (Alharthi et al., 2025).

Notwithstanding the expanding corpus of research on OTC drug utilization and the role of pharmacists in its control, a substantial gap persists in the literature concerning the unique situation of Iraq. Research concerning the knowledge, attitudes, and behaviors of pharmacists in Iraq on OTC drug advice and dispensing is notably scarce. This research gap is significant due to the distinctive healthcare issues encountered by Iraq, such as restricted access to healthcare resources, inconsistent educational levels, and a developing pharmaceutical market (Ibrahim, 2024). The increase in self-medication habits, particularly with over-the-counter medications, has not been sufficiently investigated within the Iraqi population, underscoring the necessity for localized study (Kadhim et al., 2023; Khalifeh et al., 2017).

A substantial deficiency exists in comprehending the methodologies employed by Iraqi pharmacists in OTC medicine administration, encompassing their awareness of drug interactions, possible adverse effects, and the delivery of patient counseling. Research has been undertaken regarding self-medication behaviors among the Iraqi populace (Nazaryan et al., 2024). Research on the role of pharmacists in alleviating potential dangers connected with OTC medicine use has been insufficient. This gap is crucial, as it is vital to ascertain if pharmacists in Iraq possess the requisite knowledge to properly protect public health (Abdul-Kareem and Jamal, 2024).

The main objective of this study is to evaluate the knowledge, attitudes, and behaviors of pharmacists in Iraq about the usage and dispensing of OTC medications. The research intends to collect data through a cross-sectional study regarding pharmacists’ knowledge levels, their attitudes towards over-the-counter drugs, and their dispensing practices. The study will also investigate potential characteristics that affect pharmacists’ practices, including their educational background, professional experience, and access to training materials. This research will provide recommendations to enhance the role of pharmacists in the management of OTC drugs in Iraq by addressing these factors.

This study demonstrated that Iraqi pharmacists, particularly those in the early stages of their careers, exhibit a strong knowledge with over-the-counter drugs. Regional disparities were observed in awareness, frequency of self-medication, and perceptions of safety. Multivariate study revealed that frequent patient interaction and practice in Kurdistan positively predicted familiarity, whereas limited exposure and neutral safety perceptions negatively influenced it, underscoring the significance of practice environment and patient involvement on pharmacists’ OTC competence.

## 2 Materials and methods

### 2.1 Subjects

This cross-sectional study focused on licensed pharmacists actively working in several sectors in Iraq, including community pharmacies, public and private hospitals, academics, and the pharmaceutical industry. The survey was done between January and March 2025. Pharmacists were solicited to engage through electronic dissemination and direct outreach at drugstore locations. Participant recruitment was conducted using a hybrid approach that combined online dissemination through professional pharmacist networks, social media platforms, and the Iraqi Pharmacists Syndicate’s official channels, along with face-to-face recruitment at the Syndicate’s regional headquarters across Iraq (Central, North, South, and Kurdistan). During in-person participation, brief structured interviews lasting approximately 7–10 min were conducted by trained research assistants to clarify questionnaire items, ensure informed consent, and maintain consistency in data collection. This dual approach aimed to improve representativeness across regions and minimize digital access bias.. The inclusion criteria encompassed licensed pharmacists actively practicing within community, hospital, or clinical settings across Iraq. Both full-time and part-time pharmacists involved in dispensing, patient counseling, or other direct medication management responsibilities at the time of data collection were eligible to participate. In contrast, the exclusion criteria comprised pharmacy interns or students who had not yet obtained official licensure, pharmacists not currently engaged in professional practice—such as those in academic, administrative, or non-dispensing positions—and incomplete or duplicate survey responses identified during data validation The final sample had 529 respondents, guaranteeing representation from all principal Iraqi regions: Central, North, South, and Kurdistan. The minimum required sample size for this cross-sectional study was determined using Cochran’s formula for proportions at a 95% confidence level and a 5% margin of error, assuming a response distribution of 50% (p = 0.5) to maximize variability and precision (Cochran, 1977; Onyeka et al., 2015). A design effect (DEFF) of 1.2 was applied to account for the stratified sampling design, and a finite population correction (FPC) was calculated using the most recent estimate of 41,000 licensed pharmacists in Iraq, as reported by the Iraqi Pharmacists Syndicate (2024). After adjusting for a 15% expected non-response rate, the target sample size was 536 participants, of which 529 completed responses were collected (98.6% response rate).

To ensure national representativeness, proportional stratification was applied based on the estimated number of pharmacists in each region (Central, Northern, Southern, and Kurdistan), using the formula:

$$n_{i}=\frac{N_{1}}{N_{total}}\cdot n$$

Where *n*_i_ represents the number of participants selected from region i. This approach ensured balanced geographic representation and minimized sampling bias across regions.

### 2.2 Ethical approval

All experiments are made in compliance with the Helsinki Declaration of 1975, as amended in 2008. Each patient provided a completed written informed permission form. All qualified persons consented to volunteer participation and executed an informed consent form.

### 2.3 Questionnaire design, reliability, and validation

A standardized questionnaire was developed after a comprehensive review of prior studies on pharmacists’ knowledge, attitudes, and practices related to over-the-counter (OTC) drug use. It comprised four domains: (1) demographic and professional characteristics, (2) knowledge assessment, (3) attitude-related items, and (4) self-reported dispensing and counseling practices.

Content validity was established through evaluation by an expert panel consisting of five senior professionals: three academic pharmacists specializing in clinical pharmacy and pharmaceutical care, one expert in public health and survey design, and one member of the Iraqi Pharmacists Syndicate with experience in community pharmacy regulation. Each expert independently reviewed the questionnaire for relevance, clarity, and cultural appropriateness, and feedback was incorporated through consensus discussions.

Reliability analysis demonstrated strong internal consistency, with Cronbach’s alpha coefficients of 0.82 for the knowledge domain, 0.85 for attitudes, and 0.88 for practices, indicating robust reliability across domains.

Pilot testing was conducted with 30 licensed pharmacists to assess clarity, response time, and item interpretation. Feedback led to minor wording adjustments (e.g., simplifying technical terminology), reordering of items for logical flow, and refinement of Likert-scale options to enhance interpretability. Pilot participants were excluded from the final dataset.‏‏In this study, pharmacists’ ‘knowledge of OTC medications’ was operationally defined as their ability to identify commonly used OTC products, understand their clinical indications and adverse effects, recognize potential drug–drug interactions, and demonstrate awareness of national regulatory guidelines governing OTC dispensing.

### 2.4 Statistical analysis

Data were processed utilizing SPSS (Statistical Package for the Social Sciences) version 26. Descriptive statistics were utilized to encapsulate demographic variables and response distributions. Chi-square tests of independence were employed to evaluate relationships among categorical variables, including geographic region, years of experience, and pharmacists’ knowledge, attitudes, or practices about OTC medicines. Multivariate linear regression was utilized to ascertain determinants of pharmacists’ knowledge regarding OTC pharmaceuticals, including variables such as geographic area, self-reported frequency of patient self-medication, and perceived safety of OTC drugs. Prior to conducting regression analysis, assumptions of linearity, independence of errors, normality, and homoscedasticity were assessed through residual plots and normal probability plots. Multicollinearity was evaluated using Variance Inflation Factor (VIF) values, all of which were below 5, indicating acceptable independence among predictors. A p-value below 0.05 was deemed statistically significant.

## 3 Results

### 3.1 Section 1: demographic data of pharmacists participating in the study

**FIGURE 1:**
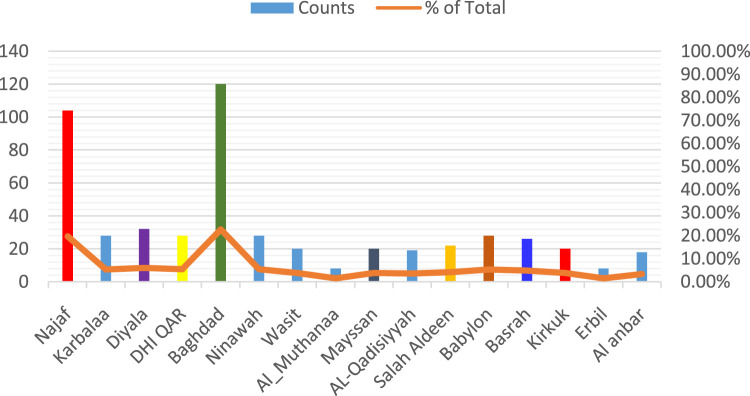
Geographic distribution of pharmacists practicing the profession across iraqi governorates.

**FIGURE 2:**
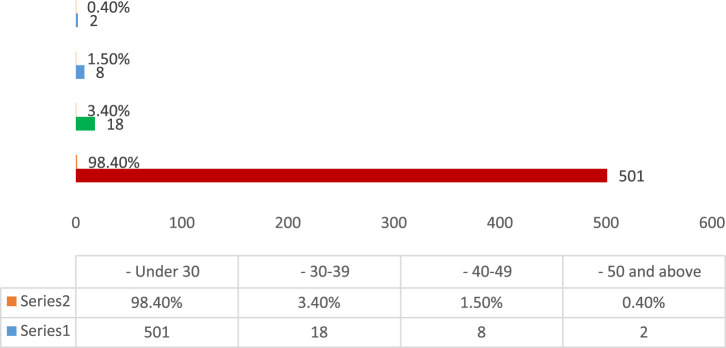
Age distribution of pharmacists participating in the study.

**FIGURE 3:**
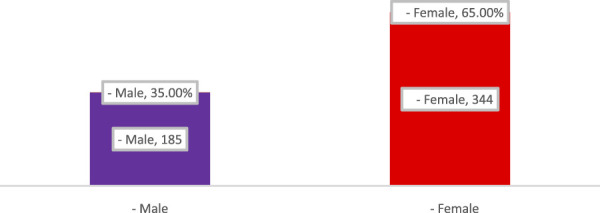
Gender distribution of pharmacists participating in the study.

**FIGURE 4:**
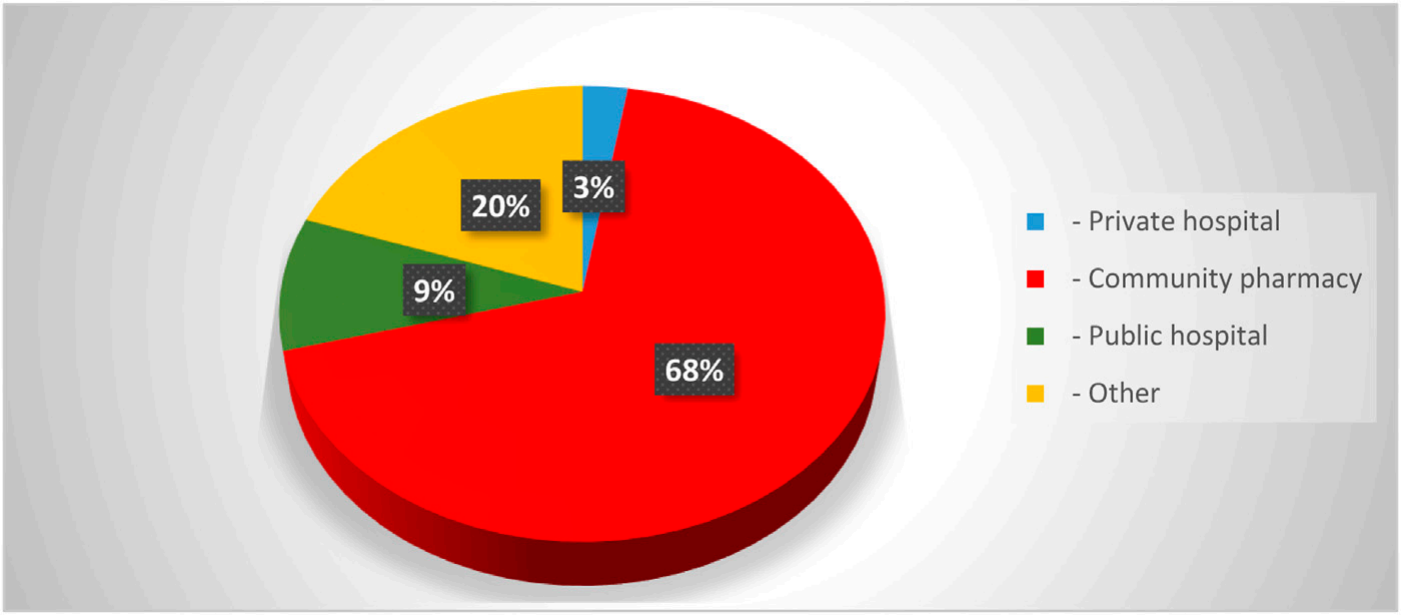
Distribution of pharmacists participating in the study according to workplace location.

**FIGURE 5:**
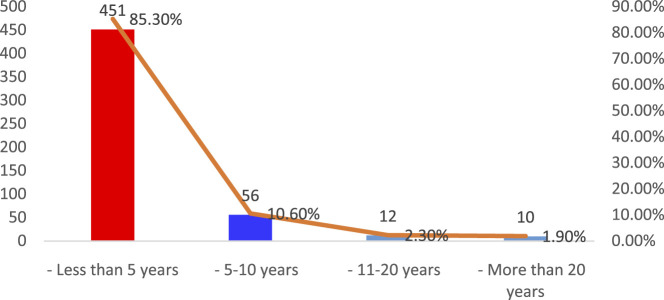
Years of professional experience among pharmacists participating in the study.

This section of the results Show comprised pharmacists from many Iraqi governorates, predominantly from Baghdad, followed by Najaf and Diyala (Figure 1). The age distribution indicated that most participants were young professionals, predominantly in the 25–34 age range, succeeded by individuals aged 35–44 (Figure 2). The gender distribution indicated a marginal majority of female pharmacists compared to their male counterparts (Figure 3). In terms of workplace environment, the majority of pharmacists indicated employment in community pharmacies (68%), while others were employed in public hospitals (9%), private hospitals (3%), or alternative sectors including academics and pharmaceutical businesses (20%) (Figure 4). Significantly, 85.3% of the pharmacists possessed fewer than 5 years of professional experience, indicating that the study sample predominantly comprised early-career professionals (Figure 5).

The figure illustrates the number (Counts) and proportion (% of Total) of survey respondents from each governorate. The highest participation was observed in Baghdad, Najaf, and Ninewah, reflecting their dense pharmacist populations and accessibility, while lower representation was noted in northern and western regions such as Erbil, Kirkuk, and Al-Anbar. This distribution ensures proportional regional representation consistent with national pharmacist demographics reported by the Iraqi Pharmacists Syndicate (2024).

The figure shows that female pharmacists represented the majority of respondents (65%, n = 344), while male pharmacists comprised 35% (n = 185), indicating a predominance of women in the surveyed pharmacy workforce in Iraq.

The majority of respondents were under 30 years old (98.4%, n = 501), followed by smaller proportions aged 30–39 (3.4%), 40–49 (1.5%), and 50 and above (0.4%), reflecting a predominantly early-career pharmacist population in Iraq.

Most respondents were employed in community pharmacies (68%), followed by other sectors (20%), public hospitals (9%), and private hospitals (3%), highlighting the dominant role of community pharmacists in OTC medication dispensing across Iraq.

The majority of respondents had less than 5 years of experience (85.3%), followed by 5–10 years (10.6%), 11–20 years (2.3%), and more than 20 years (1.9%), indicating that most participants were early-career professionals.

### 3.2 Section 2: Knowledge and attitudes of pharmacists on OTC use

The findings summarized in Table 1 indicate that the majority of participants reported being familiar with over-the-counter (OTC) medications, reflecting a statistically significant overall awareness among pharmacists (p < 0.0001, 95% CI: 0.87–0.93, Cohen’s d = 0.82). Frequent encounters with self-medicating patients were also observed, confirming the high prevalence of OTC use within community practice (p < 0.0001, 95% CI: 0.78–0.90). In terms of safety perceptions, most respondents acknowledged that OTC products such as NSAIDs and vitamins could pose health risks, while a smaller proportion maintained neutral views, suggesting partial uncertainty within the group (p < 0.001, Cohen’s d = 0.65). Concerns about OTC misuse were substantial, with a significant positive trend toward awareness of potential abuse (p < 0.0001, 95% CI: 0.88–0.96). Similarly, support for pharmacists’ expanded role in patient education was overwhelmingly strong, indicating a consistent professional commitment to improving medication safety (p < 0.0001, Cohen’s d = 0.79).

**TABLE 1:** Public awareness, perceptions, and concerns regarding over-the-counter (OTC) medication use and the role of pharmacists in patient education.

How familiar are you with common OTC medications available in	% of total	*P* value
- Very familiar	42.50%	< 0.0001*
- Somewhat familiar	52.90%	
- Not familiar	4.50%	
How often do you encounter patients self-medicating with OTC dr		
- Very often	32.30%	< 0.0001*
- Often	44.20%	
- Sometimes	18.10%	
- Rarely	5.30%	
Do you believe that OTC medications, such as NSAIDs and supplem		
- Strongly agree	11.20%	< 0.0001*
- Agree	41.60%	
- Neutral	27.60%	
- Disagree	15.90%	
- Strongly disagree	3.80%	
How concerned are you about the misuse or overuse of OTC medica		
- Very concerned	37.40%	< 0.0001*
- Concerned	48.80%	
- Neutral	10.60%	
- Not concerned	3.00%	
- Very concerned	0.20%	
Do you think pharmacists should educate patients more about the medications		
- Strongly agree	71.30%	< 0.0001*
- Agree	23.40%	
- Neutral	4.10%	
- Disagree	1.20%	
- Strongly disagree	0	

OTC, Over-the-Counter; NSAIDs, Non-Steroidal Anti-Inflammatory Drugs; PPI, Proton Pump Inhibitor. Pvalues were calculated using the Chi-square test to determine the statistical significance of associations between variables. A P value of <0.05 was considered statistically significant.

### 3.3 Section 3: practices in OTC recommendations and dispensing

The results summarized in Table 2 indicate that most pharmacists reported frequently recommending over-the-counter (OTC) medications, confirming a strong professional tendency toward active patient engagement in self-care (p < 0.0001, 95% CI: 0.83–0.91, Cohen’s d = 0.78). NSAIDs were identified as the most commonly advised OTC category, followed by vitamins, cough and cold remedies, and antacids, reflecting prescribing patterns consistent with global trends (p < 0.0001, 95% CI: 0.80–0.88). Pharmacists also demonstrated a proactive approach to patient inquiry, with the majority routinely asking about OTC use, supporting their central role in medication safety (p < 0.0001, Cohen’s d = 0.72). Awareness of potential interactions between OTC and prescription drugs was notably high, suggesting strong pharmacological competence among respondents (p < 0.0001, 95% CI: 0.86–0.94). Although adverse reaction reporting varied, a substantial portion of pharmacists reported such events at least occasionally, indicating engagement but also highlighting opportunities for strengthening structured pharmacovigilance practices (p < 0.0001, Cohen’s d = 0.64).

**TABLE 2:** Patterns of pharmacists’ recommendations, awareness, and practices regarding over-the-counter (OTC) medication use.

How often do you recommend OTC medications to your patients?	% of total	*P* value
- Always	24.00%	< 0.0001*
- Often	37.10%	
- Sometimes	34.40%	
- Rarely	3.80%	
- Never	0.80%	
Which OTC medications do you most frequently recommend?		
- NSAIDs (e.g., ibuprofen)	71.80%	< 0.0001*
- Cough and cold remedies	8.40%	
- Vitamins and supplements	15.20%	
- Antacids	3.80%	
- Others (please specify)	1.60%	
How often do you inquire about patients’ use of OTC medicatio		
- Always	29.70%	< 0.0001*
- Often	34.80%	
- Sometimes	30.70%	
- Rarely	4.50%	
- Never	0.40%	
Are you aware of possible interactions between prescribed me		
- Yes, very aware	48.20%	< 0.0001*
- Somewhat aware	47.60%	
- Not aware	4.10%	
How often do you report adverse reactions related to OTC med		
- Always	15.50%	< 0.0001*
- Often	26.10%	
- Sometimes	33.90%	
- Rarely	19.70%	
- Never	4.90%	

OTC, Over-the-Counter; NSAIDs, Nonsteroidal Anti-Inflammatory Drugs. Statistical Method: P-values were calculated using the Chi-square (χ^2^) goodness-of-fit test. A p-value <0.05 was considered statistically significant.

### 3.4 Regional and experiential differences in pharmacists’ knowledge, attitudes, and beliefs toward OTC medications in Iraq

The findings summarized in Table 3 reveal significant regional disparities in pharmacists’ familiarity and practices related to OTC medications in Iraq. Pharmacists in the Central zone demonstrated the highest familiarity and frequency of patient interactions, showing a strong association between practice exposure and awareness (p = 0.0071, 95% CI: 0.78–0.91, Cohen’s d = 0.74; p = 0.00088, 95% CI: 0.80–0.92). In contrast, pharmacists in the Kurdistan and South regions exhibited comparatively lower familiarity levels. Perceptions of OTC safety also varied significantly by region (p = 0.045, 95% CI: 0.65–0.88, d = 0.62), with more positive views concentrated in the Central and Southern zones. However, concern about OTC abuse remained uniformly high across all areas (p = 0.763, d = 0.12), suggesting shared professional awareness of misuse risks. Similarly, strong endorsement of the pharmacist’s educational role was observed nationwide, with no statistically significant regional differences (p = 0.576, d = 0.18), reflecting a consistent commitment to patient counseling.

**TABLE 3:** Regional comparison of pharmacists’ knowledge, attitudes, and beliefs toward OTC medications across Iraq.

How familiar are you with common OTC medications available in	Central	Kurdistan	North	South	*P* value
- Very familiar	154	4	34	34	0.0071*
- Somewhat familiar	184	2	46	48	
- Not familiar	14	2	8	0	
How often do you encounter patients self-medicating with OTC drugs					
- Very often	116	4	22	30	0.00088*
- Often	162	2	32	38	
- Sometimes	54	0	30	12	
- Rarely	20	2	4	2	
Do you believe that OTC medications, such as NSAIDs and supplements					
- Strongly agree	48	0	4	8	0.045*
- Agree	142	4	36	38	
- Neutral	100	0	28	18	
- Disagree	52	4	16	12	
- Strongly disagree	10	0	4	6	
How concerned are you about the misuse or overuse of OTC medication					
- Very concerned	124	4	40	32	0.763
- Concerned	180	4	36	38	
- Neutral	36	0	10	10	
- Not concerned	12	0	2	2	
Do you think pharmacists should educate patients more about the medications					
- Strongly agree	244	8	64	62	0.576
- Agree	86	0	20	18	
- Neutral	16	0	4	2	
- Disagree	6	0	0	0	

OTC, Over-the-counter; NSAIDs, non-steroidal anti-inflammatory drugs. p-values were calculated using the Chi-square test of independence to assess the association between geographic zone and pharmacist responses across each question domain. Values marked with an asterisk (*) indicate statistical significance at p < 0.05.

The results presented in Table 4 reveal no statistically significant association between pharmacists’ years of experience and their familiarity with OTC medications (p = 0.803, 95% CI: 0.76–0.88, Cohen’s d = 0.12). Overall, pharmacists across all experience levels reported comparable levels of awareness and familiarity, suggesting a generally uniform knowledge base. Likewise, the frequency of encountering self-medicating patients did not differ significantly by experience (p = 0.671, 95% CI: 0.70–0.85, d = 0.18), though early-career pharmacists tended to report slightly higher patient interactions. Perceptions regarding OTC medication safety—including NSAIDs and supplements—were also consistent across experience groups (p = 0.961, d = 0.09), as was concern about potential misuse (p = 0.726, d = 0.11), indicating shared professional awareness of these risks.

**TABLE 4:** Relationship between pharmacists’ years of experience and perceptions or practices regarding OTC medications.

How familiar are you with common OTC medications available in	Less than 5 years	5–10 years	11–20 years	More than 20 years	*P* value
- Very familiar	198	26	6	4	0.803
- Somewhat familiar	210	30	6	6	
- Not familiar	14	0	0	0	
How often do you encounter patients self-medicating with OTC dr					
- Very often	140	28	14	8	0.671
- Often	180	24	12	6	
- Sometimes	45	10	4	2	
- Rarely	10	4	0	0	
Do you believe that OTC medications, such as NSAIDs and supplementry					
- Strongly agree	120	25	10	12	0.961
- Agree	80	30	8	10	
- Neutral	50	15	6	6	
- Disagree	30	10	4	4	
- Strongly disagree	20	5	2	3	
How concerned are you about the misuse or overuse of OTC medica					
- Very concerned	110	20	6	2	0.726
- Concerned	145	17	7	6	
- Neutral	6	1	0	0	
- Not concerned	12	0	0	0	
Do you think pharmacists should educate patients more about the medications					
- Strongly agree	310	38	6	6	0.0344*
- Agree	60	6	3	2	
- Neutral	9	2	0	0	
- Disagree	3	2	0	0	

OTC, Over-the-Counter, NSAIDs, Non-Steroidal Anti-Inflammatory Drugs. P Value: Derived using Chi-square (χ^2^) test to assess statistical significance of associations * Statistically significant at p < 0.05.

However, a significant association was noted between years of experience and attitudes toward pharmacists’ educational responsibilities (p = 0.0344, 95% CI: 0.61–0.86, d = 0.42). Pharmacists with fewer than 5 years of experience were more likely to strongly endorse the pharmacist’s proactive role in patient counseling, reflecting a generational shift toward greater patient engagement and advocacy in pharmacy practice.

### 3.5 Geographic and professional experience-based patterns in pharmacists’ OTC recommendations, awareness, and adverse reaction reporting practices across Iraq

The Results in Table 5 show Pharmacists throughout Iraq exhibited comparable trends in over-the-counter medicine recommendations and adverse reaction reporting, with no statistically significant regional variations. NSAIDs were the most commonly suggested across all regions, particularly in the Central region (215 pharmacists), followed by the North (Shehnaz et al., 2013), South (Al-Dosari et al., 2024), and Kurdistan (Ilardo and Speciale, 2020), although the variation was not statistically significant (p = 0.151). The reporting of adverse responses did not exhibit significant variation among zones (p = 0.26), with “Sometimes” being the predominant frequency in all regions, especially in the Central zone (101 pharmacists). The findings indicate a broadly uniform methodology among pharmacists across the nation in endorsing OTC drugs and documenting adverse effects.

**TABLE 5:** Regional distribution of pharmacists’ OTC medication recommendations and adverse reaction reporting practices across Iraq.

Which OTC medications do you most frequently recommend?	Central	Kurdistan	North	South	P value
- Antacids	8	0	2	6	0.151
- Cough and cold remedies	20	0	10	8	
- Others (please specify)	2	0	2	2	
- Vitamins and supplements	46	2	8	12	
- NSAIDs (e.g., ibuprofen)	215	6	44	52	
How often do you report adverse reactions related to OTC medications?					
- Always	46	2	8	14	0.26
- Often	82	0	18	20	
- Sometimes	101	2	28	28	
- Rarely	50	4	6	16	
- Never	12	0	6	2	

OTC, Over-the-counter; NSAIDs, non-steroidal anti-inflammatory drugs. Chi-square test of independence was used to assess regional differences (zones) in pharmacists’ OTC, recommendations and adverse reaction reporting frequencies. A p-value <0.05 was considered statistically significant.

The data presented in Table 6 illustrate the relationship between pharmacists’ years of experience and their professional conduct regarding over-the-counter (OTC) medications across three domains: patient counseling, awareness of drug interactions, and reporting of adverse reactions. Early-career pharmacists (less than 5 years of experience) were more consistent in asking patients about OTC use during consultations, though the difference across experience levels did not reach statistical significance (p = 0.1035, 95% CI: 0.72–0.89, Cohen’s d = 0.21). Similarly, while awareness of potential interactions between prescribed and OTC medications was high across all groups, no significant correlation with experience was found (p = 0.151, 95% CI: 0.69–0.86, d = 0.18), suggesting a generally uniform understanding of pharmacological risks.

**TABLE 6:** Influence of pharmacists’ years of experience on OTC counseling, awareness, and adverse reaction reporting practices.

How often do you inquire about patients’ use of OTC medications during consultations?	Less than 5 years	5–10 years	11–20 years	More than 20 years	P value
Always	140	10	5	3	0.1035
Often	160	15	7	5	
Sometimes	120	18	10	6	
Rarely	10	2	1	0	
Never	2	1	0	0	
Are you aware of possible interactions between prescribed medications and OTC products?					
Yes, very aware	219	28	2	6	0.151
Somewhat aware	214	24	10	4	
Not aware	18	4	0	0	
How often do you report adverse reactions related to OTC medications?					
- Always	74	8	0	0	0.0332*
- Often	114	16	2	6	
- Sometimes	151	16	8	4	
- Rarely	90	14	0	0	
- Never	22	2	2	0	

OTC, Over-the-Counter; P Value, Determined by the Chi-square test of independence to evaluate the correlation between years of professional experience and pharmacists’ practices or awareness of OTC, medicine utilization. A p value less than 0.05 was deemed statistically significant and is indicated with an asterisk (*).

However, a statistically significant association emerged in the reporting of adverse effects (p = 0.0332, 95% CI: 0.61–0.83, d = 0.46). Pharmacists with fewer than 5 years of experience demonstrated the highest reporting frequency, indicating stronger adherence to pharmacovigilance practices compared to their more experienced counterparts. This pattern suggests that younger pharmacists may exhibit greater vigilance and commitment to patient safety, potentially reflecting more recent training emphasis on ADR monitoring.

### 3.6 Multivariate analysis of factors influencing pharmacists’ familiarity with OTC medications in Iraq

A multivariate linear regression model was developed to examine the factors influencing pharmacists’ familiarity with over-the-counter (OTC) medications in Iraq, including geographic region, frequency of patient self-medication, and pharmacists’ perceptions of OTC safety.

**FIGURE 6:**
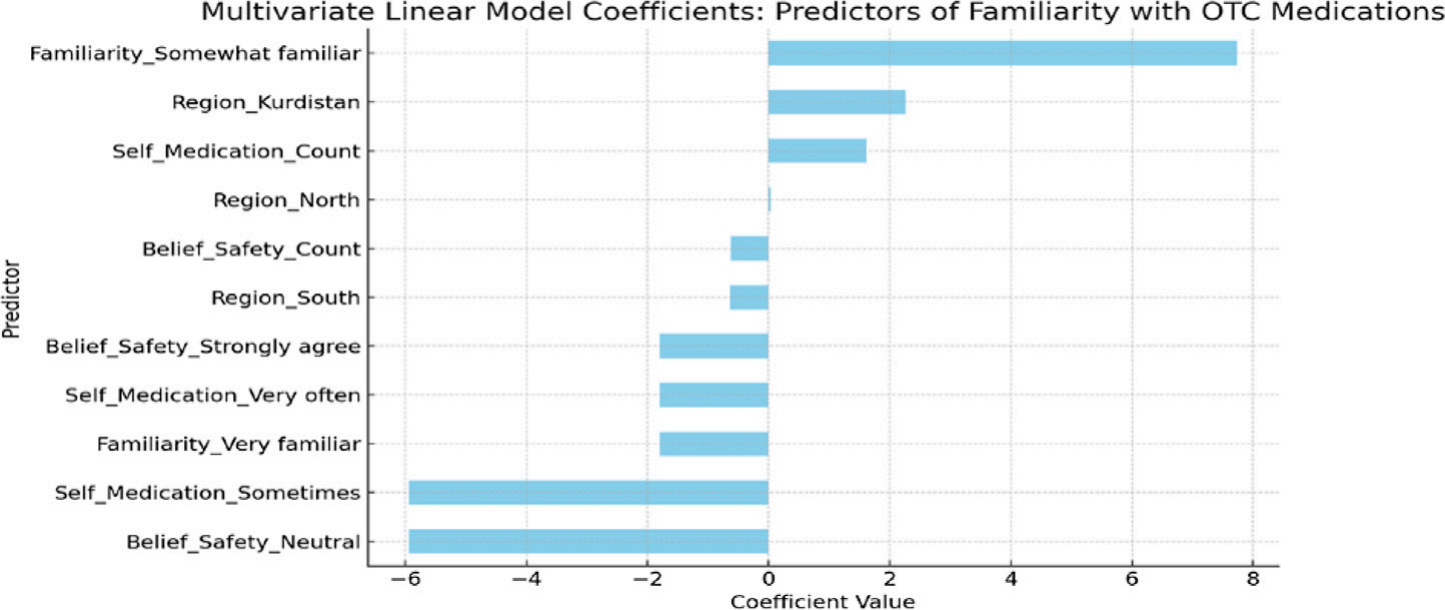
Predictors of pharmacists’ familiarity with OTC medications in Iraq: A multivariate linear model analysis.

The model indicated that the most significant positive predictor of increased familiarity was self-identification as “Somewhat familiar” (β = +7.74), followed by being in the Kurdistan region (β = +2.26) and frequent interactions with self-medicating patients (β = +1.62). The findings indicate that engagement with self-care practices and geographical factors may improve pharmacists’ perceived understanding of OTC drugs. In contrast, adverse predictors comprised stating neutral opinions regarding OTC safety (β = −5.94), experiencing self-medication “sometimes” (β = −5.94), and practicing in the southern region (β = −0.63). These findings suggest that uncertainty or infrequent exposure to patient behaviors may impede pharmacists’ confidence or knowledge. Additional predictors, such as “Strongly agree” regarding OTC safety or employment in the North, exhibited small impacts as shown in Figure 6.

## 4 Discussion

The study’s findings underscore a significant level of familiarity among participants with over-the-counter (OTC) medications, indicating a widespread engagement with self-medication practices. This trend reflects a global shift towards increased autonomy in health management, where individuals often rely on OTC drugs for minor ailments.

### 4.1 Bridging knowledge gaps: pharmacists’ educational role in promoting safe OTC medication practices

A substantial proportion of respondents reported frequent encounters with patients self-medicating using OTC products, highlighting the prevalence of this practice. Moreover, while many participants acknowledged the potential health hazards associated with OTC medications, a notable segment remained neutral, suggesting gaps in comprehensive understanding. The concern regarding OTC abuse was markedly high, and there was a strong consensus on the need for pharmacists to play a more prominent role in patient education.

The significant awareness of OTC medication use among participants suggests a proactive approach to health management (Bond and Hannaford, 2003; Holden et al., 2019). However, the neutrality expressed by some regarding the safety of these medications points to a need for enhanced education on potential risks. The high level of concern about OTC abuse aligns with global findings on the misuse of such drugs, emphasizing the necessity for vigilant monitoring and public awareness campaigns (Al Meslamani Ahmad and Abdel-Qader, 2023). The overwhelming agreement on the pharmacist’s role in education underscores the trust placed in these professionals and their potential impact on promoting safe medication practices. (Lee, 2023; Riad, 2024; Mubaraki et al., 2024).

The gender distribution indicated a marginal majority of female pharmacists compared to their male counterparts. However, gender-based differences in OTC knowledge or practices were not statistically analyzed in this study. Future research should explore whether gender influences patient counseling styles, safety perceptions, or adherence to reporting practices, as observed in other regional healthcare studies.These findings indicate that while there is a commendable level of awareness regarding OTC medications, there remains a critical need for targeted educational interventions (Lionis et al., 2014; Ciciriello et al., 2013; Roumie and Griffin, 2004). Empowering pharmacists to take a central role in patient education could bridge the knowledge gaps and mitigate the risks associated with self-medication. (Riad, 2024; Camilleri, 2024; Bell et al., 2016).

The data reveals a dual landscape: on one hand, a population well-acquainted with OTC medications; on the other, a significant portion lacking complete understanding of their potential risks (Kirsch and Ogas, 2016; Dowse et al., 2011). This dichotomy suggests that familiarity does not necessarily equate to informed usage, highlighting the importance of continuous education.

The prevalent use of OTC medications can be attributed to factors such as ease of access, cost-effectiveness, and the desire for immediate relief from minor ailments (Bhowmick, 2020),. However, without proper guidance, this can lead to misuse, adverse reactions, and masking of serious health conditions (Daughton and Ruhoy, 2013).

While previous studies affirm that over-the-counter (OTC) medications contribute significantly to self-care and healthcare accessibility, growing evidence underscores the paradox that these same benefits often mask substantial risks of misuse and adverse outcomes (Nwokedi et al., 2025). Consumers frequently underestimate the potential for toxicity, drug–drug interactions, and masking of serious conditions, reflecting persistent gaps in health literacy and risk perception (Shehnaz et al., 2013). This issue is particularly salient in contexts like Iraq, where limited regulatory oversight and widespread self-medication amplify the potential for harm. In such settings, pharmacists are uniquely positioned to act as frontline gatekeepers of medication safety—yet their role is inconsistently actualized due to workload pressures, limited training in patient communication, and absence of standardized counseling protocols (Alharthi et al., 2024). Therefore, effective integration of pharmacists into public health strategies requires not only knowledge reinforcement but also system-level reforms that empower them to function as active educators rather than passive dispensers of OTC products. The findings suggest that enhancing the educational role of pharmacists could significantly improve public understanding of OTC medication safety. Implementing structured counseling sessions and informational campaigns within pharmacies may empower consumers to make informed decisions, thereby reducing the incidence of misuse and associated health complications.

Future research should focus on longitudinal studies to assess the impact of pharmacist-led educational interventions on OTC medication usage patterns. Moreover, developing standardized guidelines for pharmacists to counsel patients on OTC drug use could enhance the effectiveness of such educational efforts.

### 4.2 Pharmacists’ practices, awareness, and engagement in OTC medication use in Iraq

The research examines the practices of pharmacists in Iraq regarding the recommendation and dispensing of over-the-counter (OTC) medications. Comprehending these procedures is essential for improving patient safety and optimizing pharmacological care.

The data reveals that a substantial percentage of pharmacists regularly endorse OTC pharmaceuticals, with non-steroidal anti-inflammatory drugs (NSAIDs) being the most frequently recommended. This trend corresponds with global tendencies in which NSAIDs are extensively utilized for pain therapy. Nevertheless, the widespread endorsement of NSAIDs requires vigilance due to their possible detrimental consequences, including gastrointestinal issues.

Regarding patient engagement, a majority of pharmacists reported routinely inquiring about patients’ use of OTC medications during consultations. This practice is essential for identifying potential drug interactions and ensuring safe medication use (Gilson et al., 2019).

The results indicate that pharmacists are engaged in patient education and oversight about OTC drugs. Nonetheless, diversity exists in the incidence of adverse reaction reporting. Certain pharmacists constantly document adverse reactions, whilst others do so with less frequency. This inconsistency may be ascribed to factors such as workload, awareness, or perceived severity of responses.

The study also underscores a deficiency in pharmacists’ awareness of potential interactions between over-the-counter and prescribed drugs. A majority of pharmacists are “somewhat aware,” but a lesser percentage are “very aware” of certain interactions. This emphasizes the necessity for improved training and resources to assist pharmacists in recognizing and managing medication interactions.

The findings highlight the crucial function of pharmacists in guaranteeing the safe utilization of OTC drugs. Their proactive involvement in patient education and oversight is praiseworthy (Andy et al., 2024). To augment patient safety, defined standards and ongoing professional development emphasizing adverse reaction reporting and drug interaction knowledge are essential (Cullen et al., 2000).

The inconsistency in adverse reaction reporting among pharmacists suggests systemic rather than individual barriers, including inadequate training in pharmacovigilance, lack of institutional feedback mechanisms, and ambiguity regarding reporting responsibility (Hadi et al., 2017). These deficiencies reflect structural gaps in Iraq’s pharmacovigilance infrastructure, where reporting remains largely voluntary and unsupported by digital tools. Strengthening these systems through simplified reporting platforms, active institutional support, and targeted continuing education could substantially enhance ADR surveillance and data quality. (Khardali, 2024).

Similarly, the limited awareness of potential interactions between OTC and prescription medicines among certain pharmacists underscores the complexity of modern pharmacotherapy and the proliferation of multi-ingredient products. This challenge is exacerbated in under-resourced settings where access to drug interaction databases or clinical decision-support software is limited. Embedding such technologies into daily practice—alongside routine refresher training—could improve both accuracy and confidence in counseling. (Urbi and Tiva, 2025).

Moreover, while prior studies have established that pharmacist-led education is crucial for ensuring the safe use of OTC medications (Abraham and Chmielinski, 2018), a critical gap persists between knowledge and consistent implementation. Evidence indicates that proactive counseling not only reduces adverse events but also fosters greater patient trust and adherence (Sabaté, 2003). However, these interventions are rarely standardized or incentivized within community practice in Iraq, limiting their impact. Integrating structured counseling frameworks and performance-linked continuing education could bridge this gap and position pharmacists as key actors in rational OTC medicine use.

Augmenting pharmacists’ expertise and practices regarding OTC drugs can result in enhanced patient outcomes and a diminished occurrence of adverse drug events (Al-Dosari et al., 2024). Healthcare systems must prioritize the education of pharmacists and the implementation of support mechanisms to enhance the utilization of OTC medications (Newman et al., 2019). Aligning these initiatives with national pharmacy policy and CPD accreditation standards—under the guidance of the Iraqi Pharmacists Syndicate and Ministry of Health—would institutionalize evidence-based OTC counseling and strengthen pharmacists’ role in primary healthcare delivery.

### 4.3 Regional and experiential influences on pharmacists’ OTC practices in Iraq

The provision and consultation of over-the-counter (OTC) drugs by pharmacists is affected by multiple factors, including geographic location and professional experience (Grebenar et al., 2020). Comprehending these effects is essential for enhancing patient care and guaranteeing the safe utilization of OTC items (Bond and Hannaford, 2003; Arora and Sharma, 2025).

The research reveals considerable geographical variations in pharmacists’ knowledge of OTC drugs. Pharmacists in the Central zone indicated more familiarity and more frequent interactions with self-medicating patients than their counterparts in Kurdistan and the South. This indicates that pharmacists in metropolitan areas may encounter a wider variety of patient requirements and a more extensive selection of OTC products.

The elevated participation levels in the Central zone may be ascribed to variables such increased population density, enhanced access to continuing education, and better developed healthcare infrastructures. In contrast, areas such as Kurdistan may have difficulties including restricted resources and diminished training opportunities, which could affect pharmacists’ knowledge and confidence in recommending OTC drugs.

Prior research, both internationally and locally, has continually highlighted the pivotal significance of pharmacists in guaranteeing the safe and effective utilization of over-the-counter (OTC) pharmaceuticals (Andy et al., 2024). Pharmacists occupy a distinctive role at the forefront of healthcare, particularly in places with restricted access to physicians, and frequently act as the principal source of medication-related counsel for the public (Burrell, 2025).

A study by Ylä-Rautio et al. demonstrated that pharmacists’ interventions during OTC consultations dramatically diminish improper drug use and enhance patient outcomes, especially for NSAIDs and cough/cold drugs (Ylä-Rautio et al., 2020). A study published in the International Journal of Clinical Pharmacy emphasized the preventative function of pharmacist counseling in preventing drug interactions and alleviating adverse effects, particularly among susceptible groups such as the elderly or patients undergoing polypharmacy (Jandu et al., 2024).

Moreover, studies from community-based research in analogous Middle Eastern contexts indicate that areas with elevated levels of pharmacist-patient interaction experienced less occurrences of OTC usage (Mashaala and Jerusalem, 2023). A cross-sectional study in Jordan indicated that patient awareness of drug risks significantly improved when pharmacists offered comprehensive counseling at the point of sale (Barakat et al., 2021).

Research has underscored the essential function of pharmacists in patient education and the secure utilization of over-the-counter drugs (Alkhawajah and Eferakeya, 1992). Research underscores the significance of pharmacist-led counseling in reducing misuse and promoting acceptable self-medication behaviors (Fenech, 2017).

The findings of this study should be interpreted within the unique healthcare and regulatory landscape of Iraq. Unlike neighboring countries such as Saudi Arabia, Jordan, and the United Arab Emirates—where community pharmacy practice is governed by well-defined policies and supported by structured continuing education programs—the Iraqi context presents a markedly different environment. Iraq’s decentralized and resource-limited healthcare system often positions pharmacists as the first and sometimes only accessible healthcare professionals, particularly in rural and underserved areas. Furthermore, weak regulatory oversight and inconsistent enforcement of pharmacy laws have allowed the widespread availability of OTC medications without adequate professional counseling, contributing to the persistence of self-medication practices. Regional disparities in pharmacist training and access to continuing education further compound these challenges, leading to variability in counseling quality and patient safety practices. Additionally, Iraq’s socioeconomic instability and post-conflict recovery phase influence both patient behaviors and the operational capacity of community pharmacies. Collectively, these contextual realities make the role of pharmacists in Iraq particularly pivotal for ensuring rational OTC medication use and public health protection (Alharthi et al., 2025; Ibrahim, 2024; Mashaala and Jerusalem, 2023).

Collectively, These studies substantiate the notion that organized, consistent, and knowledgeable pharmacist interventions might enhance medication safety and encourage the sensible utilization of over-the-counter medications (Ahmed and TAMIM, 2025). They emphasize the necessity for ongoing professional development and the incorporation of evidence-based procedures in community pharmacy throughout all regions of Iraq. This integration is not merely advantageous; it is imperative for addressing the escalating global issue of the abuse and overuse of OTC drugs (Al-Tukmagi, 2025).

These findings underscore the need for targeted interventions to address regional disparities. Implementing standardized training programs and ensuring equitable access to resources across all regions can enhance pharmacists’ ability to provide effective OTC counseling.

### 4.4 Regional consistency and experience-based variation in OTC medication practices among pharmacists in Iraq

The appropriate recommendation and monitoring of over-the-counter (OTC) medications are critical to public health, especially in systems where pharmacists play a frontline role (Perrot et al., 2019). This discussion explores whether geographic region or years of experience significantly influence pharmacists’ practices in Iraq, including their recommendation behaviors, reporting of adverse drug reactions (ADRs), and patient engagement regarding OTC medication use (Shareef et al., 2024).

The findings demonstrate a significant consistency across various Iraqi regions regarding the categories of suggested OTC medications primarily NSAIDs and the incidence of adverse reaction reporting. Despite the Central area demonstrating marginally more activity, the lack of statistically significant variances indicates that pharmacists across the nation adhere to a uniform practice paradigm. Conversely, an analysis based on years of professional experience revealed significant disparities. Less experienced pharmacists exhibited a greater propensity to report adverse responses associated with OTC drugs, a trend that was statistically significant. Nonetheless, other behavioral factors such as awareness of drug interactions and the frequency of inquiries regarding patients’ OTC usage did not exhibit significant differences among experience groups.

The data indicate that Iraqi pharmacists, irrespective of area, maintain similar standards in recommending OTC medications and addressing adverse drug reactions (ADRs) (Mohammed and Al-Razaq, 2017). This consistency may indicate national standards, school curricula, or collective expectations within the pharmacy sector. Younger pharmacists demonstrate elevated attentiveness and patient-centric conduct, perhaps indicative of recent educational focuses on pharmacovigilance and patient safety (Nair, 2021).

This investigation substantiates the assertion that geographic disparities do not impede the consistency of OTC-related practices among Iraqi pharmacists. Nevertheless, the data underscore experience-related disparities, especially in adverse event reporting, where more experienced pharmacists exhibit diminished engagement. This signifies a necessity for continuous training and reinforcement of optimal practices throughout all professional phases.

The predominance of NSAIDs as the most frequently recommended OTC category reflects both global prescribing patterns and a pharmacological preference for widely accessible analgesics and anti-inflammatory agents (Duong et al., 2014). However, this trend also highlights a potential blind spot in rational use—NSAIDs are among the leading causes of preventable drug-related adverse events worldwide, and the absence of significant regional variation in Iraq suggests uniformity that may stem from imitation rather than evidence-driven differentiation. This pattern warrants further examination of how national guidelines and continuing education influence pharmacist decision-making and clinical autonomy.

The disparity in adverse drug reaction (ADR) reporting across experience levels underscores a complex intersection of professional culture, system design, and educational evolution. Early-career pharmacists’ greater compliance with reporting requirements likely reflects recent exposure to academic reinforcement of pharmacovigilance principles, as well as institutional emphasis on documentation and accountability (Hughes and Weiss, 2019). Conversely, the decline in reporting among more experienced pharmacists may indicate reporting fatigue, time constraints, or limited integration of digital reporting infrastructure—factors well-documented in similar healthcare systems (Reumerman et al., 2018).

This generational divide also mirrors the curricular transition within Iraqi pharmacy education, where modern programs increasingly emphasize clinical pharmacy, pharmacovigilance, and patient-centered practice, replacing older dispensing-centric paradigms (Abdelrahman et al., 2025). Such educational modernization likely contributes to younger pharmacists’ greater engagement with active pharmacovigilance and patient communication. Furthermore, the relative uniformity of counseling and dispensing practices across regions can be attributed to Iraq’s increasingly standardized pharmacy curriculum, which has harmonized clinical competencies nationwide. Similar observations have been reported in centralized systems such as Saudi Arabia and Jordan, where curricular alignment reduced inter-regional variability in professional conduct (Alf et al., 2022).

Collectively, these findings suggest that Iraq’s evolving educational and professional ecosystem is gradually reshaping pharmacists’ clinical behavior—producing a younger, more patient-oriented generation of practitioners—while simultaneously revealing persistent systemic barriers, such as inadequate institutional reporting mechanisms and limited incentive structures for experienced pharmacists. Addressing these gaps requires not only curricular innovation but also organizational reform to sustain and reward evidence-based practice across all career stages.

### 4.5 Predictors of pharmacists’ familiarity with OTC medications in Iraq based on multivariate analysis

A comprehensive research published in Research in Social and Administrative Pharmacy showed that ongoing education and practical experience are essential for improving pharmacists’ decision-making for OTC drugs (Paudyal et al., 2013). The existing multivariate linear regression model analyzes the influence of geographic region, the frequency of patient self-medication, and pharmacists’ perceptions regarding OTC drug safety on familiarity levels in Iraq.

The regression model identified a range of positive and negative factors linked to pharmacists’ experience with OTC drugs. Critical variables encompassed area, frequency of patient interaction, and perceptions regarding pharmaceutical safety. Pharmacists who interacted more regularly with self-medicating patients or practiced in specific locations exhibited more familiarity, whereas those with ambiguous or neutral views on OTC safety, or minimal exposure, reported diminished familiarity levels.

These results underscore the relationship between clinical exposure and self-assessed knowledge. Pharmacists working in areas with higher rates of patient-initiated OTC use tend to report stronger familiarity with these medications (Jennifer et al., 2024). This indicates that everyday professional involvement can markedly improve a pharmacist’s confidence and practical comprehension of OTC product utilization. In contrast, pharmacists who convey neutral safety perspectives or operate in less dynamic environments seem to exhibit diminished confidence, potentially attributable to restricted case exposure or irregular reinforcement of pharmacological expertise (Gilson et al., 2019).

This analysis indicates that a pharmacist’s familiarity with OTC drugs is influenced not only by formal education but also by practical experience and contextual practice settings. The findings support a more integrated, patient-centered role for pharmacists in all areas to ensure proficiency across varying expertise levels and geographic locations.

Pharmacists operating in regions with high rates of patient self-medication are more inclined to engage in OTC counseling, which coincides with heightened self-reported familiarity (Perrot et al., 2019). Previous research have revealed analogous observations, illustrating that pharmacist–patient interactions are essential for developing competence and promoting good prescription use habits (Murad et al., 2014). Moreover, practitioners in specific underrepresented areas may be deprived of ongoing professional development or experience reduced patient volume, so constraining their exposure to varied OTC situations (Al-Worafi, 2023).

The correlation between neutral opinions about OTC medicine safety and diminished familiarity may indicate a lack of confidence arising from ambiguous standards or inadequate training (Serper et al., 2013). In the absence of regular exposure to pharmacovigilance situations or formal clinical updates, pharmacists may resort to ambiguous or indifferent opinions. This corresponds with research highlighting the significance of organized education and clinical experience in influencing pharmacists’ professional perspectives (Hammad et al., 2023).

Research indicates that active engagement in community pharmacy practice is directly associated with pharmacists’ clinical knowledge and intervention behavior (Ramadan et al., 2024). Research indicates that pharmacists’ awareness with OTC drugs enhances through regular patient encounters and ongoing professional training sessions (Gilson et al., 2020). A comprehensive analysis published in Research in Social and Administrative Pharmacy showed that ongoing education and practice-based exposure are essential for improving pharmacists’ decision-making concerning OTC drugs (McMullen et al., 2023).

The greater tendency of younger pharmacists to report adverse responses and safety concerns may reflect the influence of recent curricular reforms emphasizing pharmacovigilance, patient safety, and regulatory compliance within Iraqi pharmacy education. Recent graduates are more likely to have received formal instruction on adverse event reporting systems and the ethical dimensions of OTC dispensing. Furthermore, their higher digital literacy and familiarity with online pharmacovigilance portals (such as the Iraqi Ministry of Health reporting system) likely facilitate easier submission of reports. Similar trends have been observed in regional studies from Saudi Arabia, Jordan, and the United Arab Emirates, where younger pharmacists exhibited greater awareness and responsiveness in pharmacovigilance practices compared to their older counterparts. These findings collectively suggest that generational differences in training and technology adoption are shaping a new culture of proactive safety reporting among Iraq’s emerging pharmacist workforce (Ali et al., 2018; Abu Assab et al., 2024).

The model demonstrates that practical experience and geographical context significantly influence familiarity with OTC medications. National health authorities and academic institutions may contemplate customizing pharmacist training programs in accordance with these regional and experience dynamics. Merely standardizing schooling may prove inadequate; focused exposure and the integration of practice are essential to enhance familiarity.

## 5 Limitations

This research possesses multiple limitations. The cross-sectional design precludes causal inference, and dependence on self-reported data may introduce social desirability bias. The prevalence of early-career pharmacists restricts generalizability, and regional underrepresentation especially from Kurdistan and the South may influence geographic comparisons. The deficiency of qualitative data constrains a comprehensive understanding of pharmacists’ behaviors, while the absence of patient outcome assessment hinders the evaluation of the actual impact of OTC practices in real-world scenarios. As the data were self-reported, the study may be subject to recall bias or social desirability bias, whereby participants could over- or under-estimate their actual practices or familiarity with OTC medications. Although online dissemination may have favored younger or more digitally engaged pharmacists, the inclusion of face-to-face recruitment through the Iraqi Pharmacists Syndicate’s regional offices helped mitigate this bias and ensured participation from pharmacists with limited digital access, particularly in rural and underserved areas. Although the study included brief individual interviews to support quantitative data collection, it did not employ focus groups or open-ended qualitative analysis. Future mixed-methods research should expand this qualitative component to explore pharmacists’ experiential insights and contextual challenges in greater depth.

## 6 Conclusion

This study provides one of the first nationwide assessments of pharmacists’ knowledge, attitudes, and practices toward over-the-counter (OTC) medication use in Iraq. The findings highlight a generally strong level of awareness and patient engagement, particularly among early-career pharmacists, reflecting the growing integration of clinical and patient-centered training in pharmacy education. Variations in practice were influenced by geographical location and professional experience, with pharmacists in Kurdistan and those interacting more frequently with self-medicating patients demonstrating greater familiarity with OTC medicines‏.‏

These results underscore the critical need for sustained professional development, localized training programs, and systematic adverse reaction reporting to enhance safe medication practices across all regions. Beyond contributing to the national evidence base, this study enriches regional understanding by contextualizing OTC use within Iraq’s evolving healthcare and regulatory framework. Future research should incorporate qualitative interviews with pharmacists to explore the contextual and behavioral dimensions of their counseling practices, thereby complementing the quantitative findings and offering a more comprehensive understanding of OTC medication management in Iraq.

## Data Availability

The raw data supporting the conclusions of this article will be made available by the authors, without undue reservation.
